# TargetSpy: a supervised machine learning approach for microRNA target prediction

**DOI:** 10.1186/1471-2105-11-292

**Published:** 2010-05-28

**Authors:** Martin Sturm, Michael Hackenberg, David Langenberger, Dmitrij Frishman

**Affiliations:** 1 Institute of Bioinformatics and Systems Biology, Helmholtz Zentrum München, Ingolstädter Landstr. 1, 85764 Neuherberg, Germany; 2 Dpto. de Genetica, Facultad de Ciencias, Universidad de Granada, E-18071, Granada, Spain; 3 Bioinformatics Group, Department of Computer Science, and Interdisciplinary Center for Bioinformatics, University of Leipzig, Haertelstrasse 16-18, D-04107 Leipzig, Germany; 4 Department of Genome Oriented Bioinformatics, Technische Universität München, Life and Food Science Center Weihenstephan, Am Forum 1, D-85354 Freising-Weihenstephan, Germany

## Abstract

**Background:**

Virtually all currently available microRNA target site prediction algorithms require the presence of a (conserved) seed match to the 5' end of the microRNA. Recently however, it has been shown that this requirement might be too stringent, leading to a substantial number of missed target sites.

**Results:**

We developed *TargetSpy*, a novel computational approach for predicting target sites regardless of the presence of a seed match. It is based on machine learning and automatic feature selection using a wide spectrum of compositional, structural, and base pairing features covering current biological knowledge. Our model does not rely on evolutionary conservation, which allows the detection of species-specific interactions and makes *TargetSpy *suitable for analyzing unconserved genomic sequences.

In order to allow for an unbiased comparison of *TargetSpy *to other methods, we classified all algorithms into three groups: I) no seed match requirement, II) seed match requirement, and III) conserved seed match requirement. *TargetSpy *predictions for classes II and III are generated by appropriate postfiltering. On a human dataset revealing fold-change in protein production for five selected microRNAs our method shows superior performance in all classes. In *Drosophila melanogaster *not only our class II and III predictions are on par with other algorithms, but notably the class I (no-seed) predictions are just marginally less accurate. We estimate that *TargetSpy *predicts between 26 and 112 functional target sites without a seed match per microRNA that are missed by all other currently available algorithms.

**Conclusion:**

Only a few algorithms can predict target sites without demanding a seed match and *TargetSpy *demonstrates a substantial improvement in prediction accuracy in that class. Furthermore, when conservation and the presence of a seed match are required, the performance is comparable with state-of-the-art algorithms. *TargetSpy *was trained on mouse and performs well in human and drosophila, suggesting that it may be applicable to a broad range of species. Moreover, we have demonstrated that the application of machine learning techniques in combination with upcoming deep sequencing data results in a powerful microRNA target site prediction tool http://www.targetspy.org.

## Background

The discovery of microRNAs in 1993 [1] introduced a totally new dimension in our understanding of how gene expression is regulated. Animal and plant genomes contain hundreds of microRNA genes [2,3] that control fundamental cellular processes and are implicated in severe diseases. Incorporated into a protein complex named RISC, microRNAs perform posttranscriptional gene regulation either through perfect binding to a cis-regulatory target site in the 3'UTR that is subsequently cleaved, leading to mRNA degradation, or by imprecise binding preferably of the microRNA 5' end to a target site, leading to possibly reversible repression of protein production. While posttranscriptional cleavage is prevalent in plants, translational repression is the predominant type of regulation in animals. Our current knowledge about the function of specific microRNAs, their targeted messenger RNAs, and the exact location of binding sites is limited.

Experimental detection of microRNA target sites is a costly and time-consuming process. While recent estimates suggest that more than 50% of human protein-coding genes may be regulated by microRNAs and that each microRNA may bind to 300-400 target genes, the latest release of the TarBase database contains information on only 995 human *in vivo *microRNA-gene interactions involving 103 distinct microRNAs and 825 distinct genes, a far cry from the actual extent of microRNA targeting [2,4]. Computational prediction of microRNA/gene interactions is a valuable tool for guiding wet-lab experiments, and it remains the only option for systematic genome-wide reconstruction of the complex combinatorial picture of microRNA-mediated target binding. It is also a challenging task because of the daunting difficulty of distinguishing true microRNA-mRNA hybrids against the noisy background of millions of possible microRNA-gene combinations and, more generally, because the basic mechanisms of microRNA target recognition remain largely unknown. Over the recent years many target prediction algorithms have been developed based on different principles [see [2] for a review]. However, the two recurring parameters used by the available methods are i) the existence of a seed match (continuous base pairing between a 3'UTR and the first 6-8 bases of a microRNA 5' end), and ii) evolutionary conservation of the target site across multiple species. Utilization of these powerful constraints in prediction algorithms leads to more reliable detection of those functional duplexes containing them, but at the same time limits our ability to identify biologically relevant microRNA target sites that do not fulfill these requirements. By definition, organism-specific or simply poorly conserved sites cannot be predicted at all if a conservation filter is applied. It has also been suggested that the seed match requirement may be too stringent, and that at least a second "type" of target sites - the so called 3' compensatory target sites - exists that cannot be detected by the seed match based methods. On the other hand, many potential microRNA-target interactions that do involve conserved seed regions may be non-functional in a physiological context [5]. Furthermore, new biological insights into the mechanisms of target binding have been obtained which could be used for predicting target sites. For example, target site accessibility to the RISC complex has been suggested as an important determinant of functional interactions.

We sought to develop a computational technique free from both the seed requirement and the conservation filter and thus capable of predicting species-specific and 3' compensatory target sites. Our method, *TargetSpy*, incorporates current biological knowledge in form of multiple sequence and structure features evaluated in the framework of the objective machine-learning prediction scheme MultiBoost with decision stumps as base learner. However, since the (conserved) seed match is a strong determinant of target site detection, even though not the only one, we additionally generate predictions for sites with conserved and unconserved seed matches by post-filtering *TargetSpy *results. We carried out extensive benchmark tests of the *TargetSpy *performance in human and *Drosophila melanogaster*. Our results suggest that *TargetSpy*, although trained on mouse, achieves the same performance as the best state-of-the-art methods in *D.melanogaster*, implying that the method can be applied to a broad taxonomic range of species for which no experimentally validated target sites are known. Furthermore, on the recently published experimental human dataset, describing changes in protein synthesis mediated by microRNAs, our method shows the highest accuracy among all tested prediction algorithms.

## Results and Discussion

### Classification of prediction approaches

Current tools for predicting microRNA target sites can be grouped into three distinct classes (Table 1). Class I is constituted by those approaches that make use of neither the seed match requirement nor conservation. Class II contains all approaches that do require a seed match, but make no use of conservation. Finally, class III is for those predictions that both require a seed match and rely on conservation.

**Table 1:** Classification of microRNA target site prediction tools

Organism	Seed match not required	Seed match required	Seed match required and conservation considered
Human	RNA22 [23] TargetSpy no-seed	PITA All 3/15 [9] TargetScanS non-conserved TargetSpy seed	EIMMo [21] MiRBase Targets [24] MiRanda [6] PicTar [22] DIANA-microT [30] TargetScanS [13] PITA TOP [9] MirTarget2 [21] TargetRank [26] TargetSpy cons. seed

Fly	RNA22 [23] TargetSpy no-seed	PITA All 3/15 [9] TargetSpy seed	EIMMo [21] PicTar [22] MiRBase Targets [24] TargetSpy cons. seed TargetScanS [13]

Some methods cannot be perfectly fitted into this scheme. For example, while *miRanda *[6] does not require a perfect seed match it weights the seed region so high that on average just around 7% of all predicted target sites show mismatches to the 7-mer seeds (microRNA nucleotides 1-7 or 2-8). *miRBase Targets *[7] uses *miRanda *for candidate generation, and permits a single mismatch to the seed region. Since both approaches additionally require the target site to be conserved, we consider them as members of class III.

Note also that *TargetSpy *generally belongs to class I, since our model does not impose a strict seed match requirement and does not consider conservation of target sites. However, we can easily build subsets of our predictions that satisfy the criteria of class II and III. Throughout this work we refer to the subset of *TargetSpy *predictions containing a perfect 7-mer seed match as *TargetSpy seed*. Likewise, *TargetSpy conserved seed *denotes a set of predicted target sites containing a conserved seed match (see *Methods *for details).

### Computational pipeline for predicting microRNA target sites

Our intention here is to build a pipeline for predicting microRNA target sites based on the multiple features described in the *Methods *section. At run time *TargetSpy *takes two multiple FASTA files as input; one with the 3' UTR sequences and the other one with the mature microRNA sequences. Note that no other extrinsic information, such as evolutionary conservation needs to be provided.

**Figure 1:**
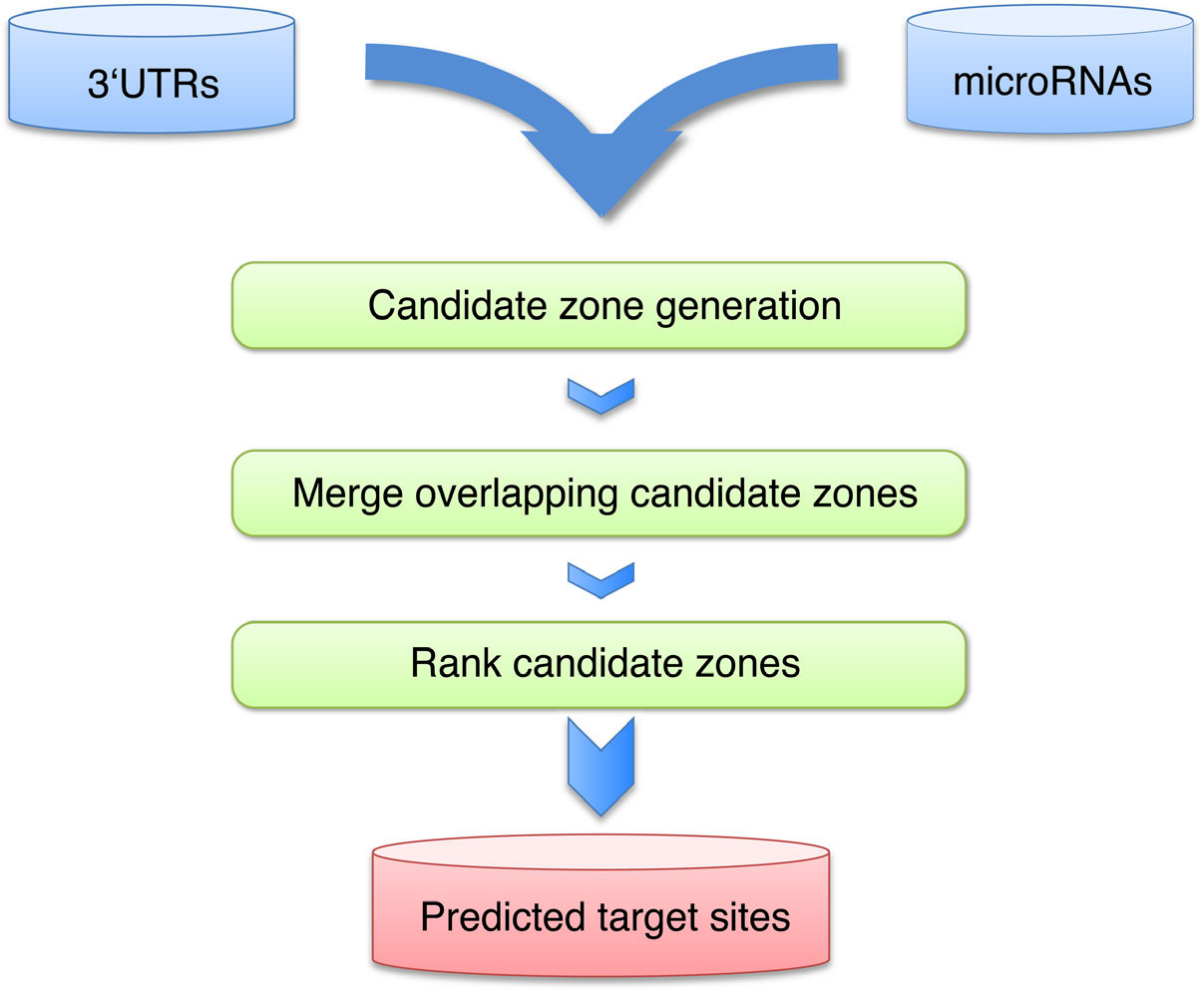
**A schematic overview of the *TargetSpy *prediction pipeline**. Annotated 3'UTR sequences and all known microRNAs from a given species serve as input. MicroRNAs are matched against 3'UTRs to generate potential candidate zones. The resulting candidate zones are classified and ranked according to their score, with overlapping zones being merged together.

For each input microRNA *TargetSpy *identifies candidate zones (stretches of RNA sequence potentially harboring a target site) in all 3'UTR sequences. It calculates the score for the representative of each candidate zone, merges overlapping candidate zones, and ranks the predictions according to their scores (Figure 1, see *Methods *for details). Using this protocol target sites were predicted for *Homo sapiens*, *Mus musculus*, *Rattus norvegicus*, *Gallus gallus *and *Drosophila melanogaster *(Table 2).

**Table 2:** Number of target sites predicted in each species by different versions of the *TargetSpy *method. See *Methods *for more detail

				Number of predicted target sites			
	Number of 3'UTRs	Average 3'UTR length	Number of microRNAs	TargetSpy no-seed sens	TargetSpy no-seed spec	TargetSpy seed sens	TargetSpy seed spec
Human	26161	1210	692	4837 k	1023 k	829 k	339 k
Mouse	18694	1082	513	1906 k	407 k	340 k	137 k
Rat	11859	760	292	535 k	113 k	91 k	36 k
Chicken	3676	927	443	372 k	80 k	59 k	24 k
Fly	15884	471	147	247 k	54 k	50 k	20 k

### Target site candidates

A usual starting point of a prediction workflow is the search for perfect seed matches in the 3'UTR of transcripts of interest. Since our goal is to develop a model that does not rely on the presence of a seed match we had to redefine the rules for selecting initial candidate target sites. Following the reasoning that a functional site is more attracted by the loaded RISC complex than its surrounding area, we identify candidates by searching for areas in the target sequence where the predicted Gibbs free energy of the microRNA-target duplex is below a certain microRNA-specific energy threshold (see *Methods *for detail). To ensure a high coverage of functional binding sites we have chosen a conservative cut-off. With this candidate definition at hand, we identified about 150 million target site candidates for all microRNAs in human.

### Selection of informative features and classifier evaluation

As described in the *Methods *section we evaluated a wide range of target site features by applying the ReliefF [8] technique (see Table 3 for the ranked list of features). Some features generally considered to be highly relevant for target site recognition by microRNA, such as the number of baseparings to the microRNA seed, performed very well. On the other hand, the feature *accessibility *with 3 nt upstream and 15 nt downstream flankings, reported in [9] to be strongly discriminative, was evaluated as poorly performing. To analyze whether this is due to the chosen flanking sequences, we tested other flanking settings and found 30 nt upstream and 30 nt downstream to perform slightly better than the 3/15 setting; however the improvement was marginal (data not shown). Interestingly, the feature *compactness *(combining the length of the target site and the number of nucleotides binding to the microRNA, see *Methods*), introduced in this work performs among the best.

**Table 3:** A ranked list of all features used in this work. The score is calculated by the ReliefF method

Rank	Features	Score
1	Number of base parings to the microRNA 8-mer seed	0.03175

2	G+C content of target site	0.01263

3	Number of base pairings to the first 8 nucleotides of the microRNA 3' end	0.01038

4	Number of consecutive base-pairings to the microRNA 3' end with two allowed non-pairing positions	0.00995

5	Occurrence of CpG in target site	0.00799

6	G+C content ratio between the microRNA and the target site	0.00642

7	Compactness	0.00619

8	T9 anchor	0.00556

9	Longest stretch of consecutive base-pairings in the hybrid	0.00513

10	Number of bulges in the microRNA of size three	0.00498

11	T1 S/W anchor	0.00491

12	Total number of base-pairings	0.00475

13	Number of bulges on the target site of size seven or greater	0.00442

14	T1 anchor	0.00434

15	Number of bulges in the microRNA of size two	0.00433

16	Occurrence of CpG in the upstream flanking area	0.00383

17	Number of bulges in the target site of size one	0.00374

18	Total bulge length of the target site	0.00362

19	Length of the target site	0.00336

20	Total bulge length of the microRNA	0.00334

21	Target site position within the 3'UTR	0.00333

22	Number of symmetric bulges	0.00290

23	G + C content upstream of the target site	0.00287

24	Number of bulges on the target site	0.00286

25	Length of the second largest bulge on the target site	0.00268

26	Mean length of bulges on the target site	0.00263

27	T9 S/W anchor	0.00261

28	Binding asymmetry	0.00255

29	Number of bulges in the target site of size two	0.00240

30	Total number of G:U wobble base pairs	0.00227

31	Local RISC accessibility 30/30	0.00220

32	Local RISC accessibility 3/15	0.00215

33	Number of bulges in the target site of size four	0.00210

34	Difference in G+C content between the first and the last nt of the target site	0.00201

35	Occurrence of CpG in downstream flanking area	0.00179

36	Number of bulges in the microRNA of size one	0.00179

37	Length of the second largest bulge on the microRNA	0.00174

38	Number of bulges on the microRNA	0.00153

39	Number of bulges in the microRNA of size five	0.00128

40	Number of bulges in the target site of size three	0.00113

41	Difference in G + C content between the target site and the 20 nt upstream and downstream flanking region	0.00112

42	Number of bulges in the target site of size five	0.00100

43	Number of bulges in the microRNA of size four	0.00084

44	G+C content downstream of the target site	0.00084

45	Number of bulges in the target site of size six	0.00021

**Figure 2:**
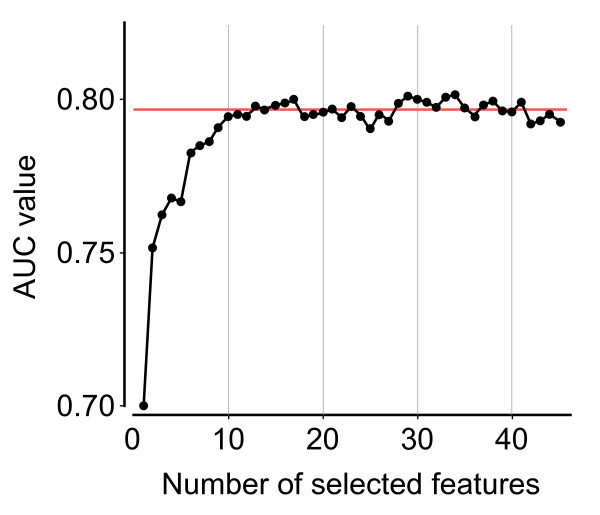
**Classifier performance as a function of the feature set size**. The classifier was evaluated in an iterative process where one feature was added at a time. Features were selected according to the ranked feature list (see Table 3), beginning with the best feature. In black the AUC values (y-axis) for the corresponding feature set size (x-axis) are shown. The red line indicates the AUC value of the feature set that was achieved by the feature subset selection approach.

Subsequently we evaluated the performance of the classifier with respect to the features used for training. We started with the single best feature and incrementally added features from Table 3 one by one, according to the ranking. Each classifier was evaluated on the training set by a standard 10-fold cross-validation procedure, as implemented in WEKA [10]. Figure 2 shows the number of features used for building the classifier and the corresponding area under the curve (AUC) values. Apparently, the AUC value increases up to the 14th added feature. Upon adding further features the performance begins to oscillate around the AUC value 0.79 and does not improve further. Since ReliefF only considers one feature at a time and does not take into account the correlation between features, we additionally applied the *Correlation-based Feature Selection *(CFS) [11] for identifying the best feature subset. This approach returned an AUC value of 0.79 (Figure 2, red line) with a set of only seven features, namely compactness, G+C content ratio between microRNA and target site, length of the longest stretch of consecutive base-pairings anywhere in the hybrid, binding asymmetry, G+C content of the target site, number of base-pairings to the microRNA 8-mer seed, and the position of the target site in the 3'UTR. The first four features are used here for the first time, while the latter three have been proposed before [12-15]. Interestingly, several of these features evaluated individually are classified merely as weak performers, while in combination the classifier exploits synergetic effects between features, making it the smallest set of all with a comparable performance. Following the common practice of selecting from all competing models of equal performance the simpler one, we chose the feature set generated by CFS for our machine learning technique.

Since *TargetSpy *tries in the first step to identify as many potential target sites as possible and subsequently ranks those according the classifier score, enormous amounts of target sites are produced from which only a fraction, the top predictions, are of interest. In order to make application and benchmarking of *TargetSpy *more transparent, we created a subset of predictions with high sensitivity and high specificity. The recognition thresholds were set in a way that the target sites with a false-positive rate lower than 5% (as evaluated in a 10-fold cross-validation) were assigned to the sensitive subset and those with a false-positive rate of 1% or less to the specific set (Table 4).

**Table 4:** Applied thresholds and limitations on the prediction subsets

Prediction dataset name	Seed match required	Conservation considered	False-positive rate threshold
TargetSpy no-seed sens	No	No	0.05
TargetSpy no-seed spec	No	No	0.01
TargetSpy seed sens	Yes	No	0.05
TargetSpy seed spec	Yes	No	0.01
TargetSpy cons. seed sens	Yes	Yes	0.05
TargetSpy cons. seed spec	Yes	Yes	0.01

### Evaluation on experimentally verified data

We next set out to evaluate the quality of the learning scheme implemented in *TargetSpy *on experimentally verified data and to benchmark *TargetSpy *against commonly used methods. This task is challenging as published methods are based on different principles, which makes it hard to compare them in a fair fashion. Our current knowledge about microRNA target sites is almost exclusively drawn from a handful of experiments exploring the targeting of a minority of the most highly expressed microRNAs [16]. These experiments may have a strong selection bias in that they usually analyze the impact of microRNA overexpression or depletion on conserved molecular mechanisms. In addition, experimentally identified targets are often biased towards computational prediction approaches used to identify the initial pool of candidates [17].

Recently the impact of microRNA overexpression and knockdown was analyzed in large-scale proteomic studies [16,18] not suffering from the selection bias discussed above. A further advantage is that none of the prediction approaches were trained on these data. Beside the low number of measured microRNAs (five) and the fact that the precise location of the target site in the respective transcript is not determined in pSILAC, until high quality deep sequencing data such as the HITS-CLIP from [19] become available in large amounts, these data constitute the current gold standard. Hence we use the fly dataset [9,17] and the human pSILAC dataset [18] for evaluation.

#### Performance comparison in *Drosophila melanogaster*

In 2005 Stark et al. [17] conducted a broad comparison of widely used target prediction approaches. A set of 133 experimentally tested functional and non-functional microRNA-gene interactions was compiled, from which 120 (functional: 61, non-functional: 59) were used for the actual comparison [17]. This dataset served as the standard of truth to evaluate the evolutionary approach to microRNA target prediction published by Gaidatzis et al. [20] and was later extended to 190 interactions by Kertesz et al. [9]. Note that this latter set also includes the 13 interactions that were excluded by Stark and colleagues since their respective 3'UTRs were not annotated. To assess the predictive power of our method and compare it with other methods, we applied it first to the original set and then to the extended set of targets. Since we assign each candidate zone a score, we are able to quantify the performance by a receiver operating characteristic (ROC) curve, making the comparison to other approaches more transparent.

**Figure 3:**
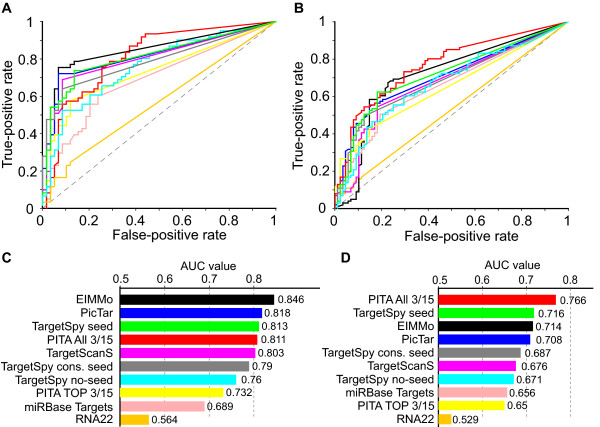
**Performance comparison of target prediction approaches**. A) and C) refer to the dataset compiled by Stark et al. [17]). B) and D) refer to the dataset compiled by Kertesz [9]. A) and B) show the ROC curves of the tested approaches, C) and D) the AUC values. The gray line indicates the performance of random guessing.

When comparing the performance of methods on the original dataset of Stark et al. (2005) (Figure 3A,C) EIMMo [21] achieves the best results, showing a high true-positive rate coupled with a low false-positive rate. Then follow PicTar [22], *TargetSpy seed, *PITA ALL 3/15 [9], TargetScanS [13] and *TargetSpy conserved seed *in the order of decreasing AUC values. The next best approach is *TargetSpy no-seed*, followed, with significant distance, by miRanda [6] and finally RNA22 [23], that is performing marginally better than random.

Despite the benefit of being able to compare all methods by just one value, looking at the ROC curve progression is even more enlightening, especially for those methods that are clustered closely together by the AUC value. Particularly interesting is the characteristic of the curves at low false-positive rates as for many experiments the amount of samples may be strongly limited. *TargetSpy conserved seed *shows the lowest false-positive rate (FPR) in the test up to a true-positive rate (TPR) of 48%. *TargetSpy seed *shows the second lowest FPR, but offers slightly better TPR, comparable to that of PicTar. Note that *TargetSpy no-seed *shows a performance that is close to class II and III methods, especially for its top predictions that cover more than 50% in TPR.

Benchmarking on the extended set of 190 experimentally verified microRNA-target interactions (see *Methods*) produces several interesting observations (Figure 3B,D). First, the AUC values are generally lower compared to the original set. Second, PITA, specifically fitted to this set, is far ahead of all other approaches. Third, the ranking of the other approaches has not changed except that i) *TargetSpy seed *performs ahead of PicTar and EIMMo, *TargetSpy conserved seed *outperforms TargetScanS and miRBase Targets [24] performs better than PITA TOP 3/15 and ii) the relative distance between *TargetSpy no-seed *and TargetScanS is reduced. Finally, the specificity in particular that of EIMMo and TargetScanS, suffered strongly especially for their top predictions.

In summary, the evaluation on experimental fly data suggests that *TargetSpy*, which was trained on mouse data, performs as good as current state-of-the-art algorithms when enforcing the seed match criterion. Furthermore, the no-seed prediction is notably better than RNA22, the other tested algorithm that does not require a perfect seed match.

#### Evaluation on pSILAC data

Selbach et al. [18] performed a comparison of the most widely used approaches by measuring the fraction of predicted target sites associated with proteins that are more strongly down-regulated than -0.1 log_2 _fold change. They generated two background (random) sets: i) a set where all mRNAs present are considered as targets, and ii) a set of all mRNAs that have a 6-mer seed match in their sequence, further referred to as the *Selbach background*. We further generated a 7-mer seed background for class II as PicTar and TargetSpy require perfect 7-mer seed matches. In order to compare class III predictors to the random expectation, we additionally introduced a background dataset for conserved 7-mer seed matches as proposed by [2]. Specifically, we searched for 6-mer (positions 2-7) seed matches that are perfectly conserved in human, chimp, mouse rat and dog that show additionally a match to either base 1 or 8 [25]. This way we have background sets produced by trivial prediction strategies for each of the three classes of prediction tools.

**Figure 4:**
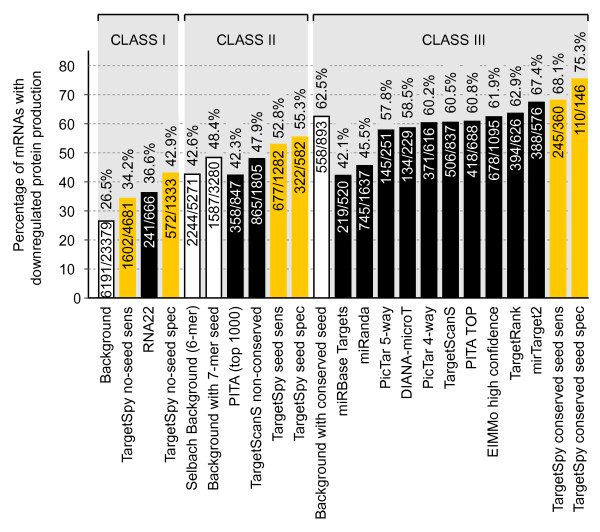
**Performance evaluation of various prediction approaches on the pSILAC data set**. This set contains changes in protein production caused by the five microRNAs miR-1, miR-16, miR-155, miR-30a-5p and let-7b. The first value in each bar represents the number of predicted microRNA-target interactions that are associated with down-regulation (log_2_-fold change < -0.1) and the second value reports the total number of interactions predicted for the pSILAC set. The value on top of each bar displays the accuracy. White bars with black outlines display the trivial predictors, *TargetSpy *is represented in orange and other approaches are displayed in black.

As seen in Figure 4 for the first class (no seed/no conservation) a completely random selection of target sites would yield a ~27% intersection (background accuracy) with down-regulated proteins in pSILAC. Both *RNA22 *and *TargetSpy no-seed *perform better than random. *TargetSpy no-seed *attains the accuracy of 34.2%, a significant improvement compared to random predictions. RNA22 shows 36.2% accuracy, however at a sensitivity that is more than 6.6 times lower than *TargetSpy no-seed sens*. In the specific setting *TargetSpy no-seed contains *still more than 2.3 times as many target sites as RNA22, but achieves an accuracy of 42.9% and is thus on the same level as the 6-mer seed background of class II.

The background accuracy for class II is at 42.6% when using 6-mer seeds (Selbach background). As PITA covers all target site candidates with seed matches beginning at the size of 6 nt and subsequently ranks them according to their accessibility, it is necessary to consider only its top ranking predictions. Following Selbach et al. 2008 [18] we took the top 1000 predictions per microRNA and found a 42.3% overlap with down-regulated proteins demonstrating an accuracy below the background level. TargetScanS, predicting non-conserved target sites with at least a 6-mer seed match, shows a higher accuracy (47.9%) than PITA and noticeably out-performs the 6-mer seed background, although it does not pass the accuracy of the 7-mer seed background. For 7-mer seeds, which are used by *PicTar *and *TargetSpy seed*, the corresponding background accuracy was 48.4%. Both *TargetSpy *seed *sens *and *TargetSpy seed spec *perform clearly better than this trivial prediction, showing accuracies of 52.8% and 55.3%, respectively, and thus perform best in class II.

The final class of target prediction approaches (seed/conservation) shows an overlap of 62.5% with random predictions. Both *miRanda *and *miRBase Targets *predictions include a small fraction of conserved target sites with imperfect seed regions, and the corresponding accuracies (45.5% and 42.1%, respectively) are below the background and close to that of the trivial seed predictor of class II. *PicTar 4-way *(those predictions where the seed match is conserved among 4 species) reaches an accuracy of 60.2%, which is also below the background. Interestingly the more stringent PicTar 5-way that additionally requires conservation in chicken, performs worse that PicTar 4-way. Also DIANA-microT, TargetScanS, PITA TOP, an official subset of PITA with conserved 8-mer seed matches required, and EIMMo with the high confidence setting (score > = 0.5) are performing slightly below background. The first approach performing above the trivial predictor of class III is TargetRank [26] (62.9%), followed by mirTarget2 [21] (67.4%) and the sensitive subset of *TargetSpy conserved seed *(68.1%). Finally, *TargetSpy conserved seed spec *achieves the highest accuracy of all methods (75.3%). It should be noted, however, that although *TargetSpy *achieves superior performance in terms of accuracy and sensitivity in classes I and II, the sensitivity of *TargetSpy *in class III is lower compared to other approaches. The higher sensitivity of some approaches might be attributed to the choice of seed match that is enforced. EIMMo, for example, integrates several different seed match definitions (including also short 6-base long ones) and shows a sensitivity that is 2.7 times higher than our approach at the sensitive threshold and more than 6 times higher when compared to our specific setting.

**Figure 5:**
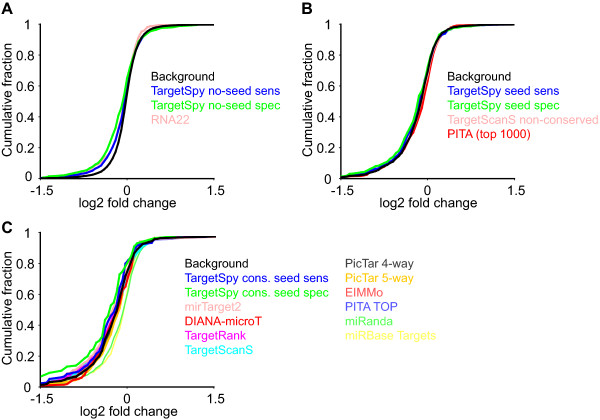
**Cumulative fraction of predicted target sites of down-regulated proteins according to the measured fold change**. The distributions are given for A) approaches not requiring a seed (class I), B) approaches requiring a seed (class II), and C) approaches requiring a seed and considering site conservation (class III).

To exclude the possibility that the performed evaluation is only valid for the chosen threshold of -0.1 log_2 _fold change, we also investigated the cumulative fraction of predicted target sites as a function of the protein log_2 _fold change. Figure 5 shows the distributions for each of the three classes. It becomes apparent that the relative performance of computational approaches remains practically unchanged for each fold change value in each class. However, as seen in Figure 5c, the advance of *TargetSpy conserved seed spec *(green line) is particularly pronounced for low log_2 _fold change values. Since low fold change values correspond to stronger protein down-regulation this may imply that our approach performs even better for highly efficient target sites.

In general our results on the pSILAC data suggest that *TargetSpy *performs best in each class, showing furthermore a constant gain in accuracy from the sensitive to the specific threshold. This finding implies that the prediction quality increases with the score and therefore the ranking of target sites imposed by the score of our model seems to have biological relevance. Under the assumption that all pSILAC interactions that are not down-regulated are true negatives, ROC plots (see Additional File 1, one for each class) confirm the performance evaluations based on accuracy.

Finally, we determined the number of functional target sites (down-regulated proteins) not possessing seed regions, which have been correctly predicted solely by *TargetSpy *but not by any other algorithm. To avoid putatively spurious seedless target sites, we excluded from consideration those gene-microRNA interactions that showed at least one perfect seed match. After removing such gene-microRNA interactions we obtained 564 unique target sites without seed region in the sensitive set and 134 in the specific set. This means that *TargetSpy *reports on average between 26 (spec) and 112 (sens) functional target sites showing no seed match per microRNA that could not be detected by any other tool.

## Conclusion

In summary, we have developed a novel computational approach for predicting microRNA target sites that neither implies the existence of a seed nor utilizes phylogenetic footprinting. Instead of using rigid rules and/or arbitrarily selected target site features, we objectively derived a set of discriminative features to be used for machine learning. Due to these important advantages *TargetSpy *i) is able to predict species specific (*i.e. *unconserved) target sites, ii) is suitable for processing poorly conserved/low quality genomic sequences for which methods that rely on conservation and species specific information will not work, and iii) allows analyzing differences in microRNA targets between various species.

We grouped computational prediction approaches into three classes, depending on their usage of a seed match criterion in order to provide a comparison of their performance among each other and against the background level. On an experimentally derived microRNA-target interaction set in *Drosophila*, our method is on par with the best available approaches. In a further benchmark on human microRNA-target data generated by the pSILAC technology, *TargetSpy *not only reported the highest accuracies in class I, but also in the other two classes for which our predictions where post filtered according to the class definition. Given that *TargetSpy*, trained on experimentally derived Ago binding sites in the 3'UTR of mouse transcripts, showed very good performance when evaluated in fly and human, we suggest that our algorithm can be applied to a broad taxonomic range of organisms.

Finally, we have shown that even on a small, high-quality data set of microRNA binding sites, derived by a deep sequencing experiment [19], machine learning techniques show high potential in the prediction of microRNA target sites. We assume that advances in this direction will become even more pronounced as more data of this kind become available.

## Methods

### Dataset of 3' UTR sequences

We retrieved 3'UTR sequences from the UCSC Genome Database [27] using the UCSC Table Browser. For human (hg18, March 2006), mouse (mm8, July 2007), rat (rn4, November 2004) and chicken (galGal2, May 2006) we used the RefSeq Genes Track, for fly (dme, April 2006) we took the FlyBase annotations. For generating target site predictions considering conserved seeds, we used Galaxy [28] to extract 3'UTR alignments for human, chimp, mouse, rat and dog from the 17-way human whole genome alignment and *D. melanogaster*, *D. yakuba*, *D. ananassae*, and *D. pseudoobscura *from the 15-way *D. melanogaster *whole genome alignment.

### Dataset of MicroRNA sequences

All mature microRNA sequences originate from the miRBase, release 12 [24]. In total we retrieved 692 microRNAs for human, 513 for mouse, 443 for chicken and 147 for fly.

### Target site predictions by previously published methods

The target predictions of *PicTar *[22] were downloaded from the UCSC database using the Table Browser and were migrated from hg17 to hg18 by applying the UCSC command line tool *liftover*. We used the predictions conserved in human, mouse, rat, chimp and dog (4-way) as well as the predictions additionally conserved in chicken (5-way). For fly we downloaded the sensitive prediction set (S1) of PicTar that is composed of predictions conserved in *D. melanogaster*, *D. yakuba*, *D. ananassae*, and *D. Pseudoobscura*, also via the UCSC Table Browser. Predictions for the human genome made by *miRanda *[6], release September 2008, were downloaded from http://microRNA.org[29]. Only predictions for transcripts contained in the RefSeq database were considered. Human and fly predictions made by *miRBase Targets *[7], version 5, were downloaded from http://microrna.sanger.ac.uk/targets/v5/. *RNA22 *[23] predictions for human 3'UTR sequences were downloaded from http://cbcsrv.watson.ibm.com/rna22.html. Since these predictions were made using Ensembl transcripts, we mapped the predictions to RefSeq genes by applying mapping tables provided by Ensembl and UCSC. Predictions of *PITA *[9] were downloaded from http://genie.weizmann.ac.il/pubs/mir07/mir07_data.html. We utilized the "*TOP*" and the "ALL" set with 3/15 flankings. *TargetScanS *[13] predictions and the corresponding microRNA family mapping table were downloaded from http://www.targetscan.org/cgi-bin/targetscan/data_download.cgi?db=vert_50. Predictions made by Gaidatzis et al. [20] were downloaded from the EIMMo server http://www.mirz.unibas.ch/. Targets predicted by *mirTarget2 *(version 3) [21] were downloaded from http://mirdb.org/miRDB. Human target site predictions of *DIANA-microT *v3.0 [30] were retrieved via the web server at http://diana.cslab.ece.ntua.gr/microT/ for the thresholds loose (score = 7.3) and strict (score = 19). Finally, we downloaded the human target site predictions of TargetRank [26] from http://hollywood.mit.edu/targetrank/.

### Experimental data

Two sets of experimentally verified target sites were used to benchmark target prediction algorithms. For evaluation on *Drosophila melanogaster*, we used the 120 experimentally tested microRNA - gene interactions compiled by Stark et al. [17] (see *Additional File *2) and the 190 interactions published by Kertesz et al. [9] (see *Additional File *3). The former set is composed of 61 functional and 59 non-functional interactions; the latter set consists of 102 functional and 88 non-functional interactions. The appropriate 3'UTR sequences were derived from the FlyBase annotations provided by UCSC. Transcripts for which no 3' UTR was available were discarded. For evaluation on human, we used an experimental dataset (see *Additional File *4) that is based on the pSILAC technique and reveals fold changes in protein production caused by five selected microRNAs [18], downloaded from http://psilac.mdc-berlin.de.

### Free energy estimates

All duplex structures and energy estimates were calculated by the RNAduplex and RNAcofold programs from the Vienna package version 1.6.1 [31]. We applied the option -noLP to exclude base pairs, which can only occur as lonely pairs and the option -e to retrieve all suboptimal structures instead of just the one with the minimum free energy. The minimum free energy that can be observed for a microRNA is defined as the energy value calculated for the duplex of the microRNA and its perfect reverse compliment.

### Generation of candidate zones

The microRNA - mRNA interaction is typically characterized as an interval within the mRNA sequence that is almost perfectly reverse complementary to the microRNA sequence over a substantial fraction of the microRNA's length, or at least over a seed region of 6-8 bases. In this work we investigate the possibility to abandon the strict requirement for the presence of a seed region and attempt to find zones of high attraction between the microRNA and its target mRNA independent of seed occurrence. Such candidate zones cover not just a particular binding site, but a larger stretch of sequence including several potential adjacent binding sites.

**Figure 6:**
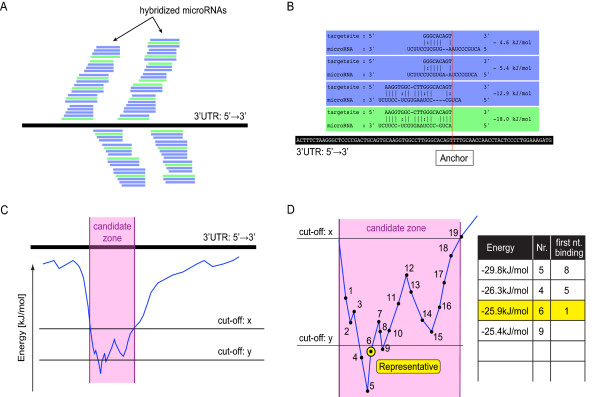
**Schematic illustration of the candidate target site generation pipeline**. A) MicroRNA - mRNA duplexes sharing the same anchor position on the mRNA are grouped. Duplexes with the lowest free energy in each group are shown in green color, all others in blue. B) Zoom-in at one group. The anchor of each hybrid (red vertical line) is the first nucleotide of the target site base-pairing with the 5' end of the microRNA. Only the energetically most favorable hybrid, shown in green, is retained for further analysis. C) Smoothed attraction graph of all the retained hybrids. A candidate zone is defined as the stretch of the target sequence (shown in purple) where the smoothed hybrid free energy falls below a certain energy threshold. D) For each candidate zone the energetically most favorable hybrid that shows base pairing within the first two nucleotides counting from the microRNA 5' end is selected as its representative.

This approach involves four subsequent steps illustrated in Figure 6A-C. First, for a pair of microRNA and mRNA sequences, all possible duplex structures predicted by RNAduplex are ordered according to the sequence position of their anchor (see next section). In a second step energy values of the selected duplexes are plotted against the respective anchor positions, resulting in a graph reflecting the attraction of individual areas of the mRNA towards the particular microRNA under study, measured in terms of Gibbs free energy values. To reduce local fluctuations the curve is smoothed by taking the average of the energy values for the current position and for its two immediate neighbors (*i.e. *by using a sliding window of length 3). In the next step those mRNA areas with a particularly strong attraction for a given microRNA are identified based on the requirement that all energy values of predicted duplex structures be below a certain cut-off *x*, and at least one duplex, we call it the representative, be below a cut-off value *y*. Based on the current experimental knowledge [32] base pairing for the representative is additionally required to start with the microRNA's first or second nucleotide counted from its 5'end. We call the areas satisfying these conditions candidate zones. The variables *x *and *y *are expressed in terms of the ratios between the observed energy of a duplex and the maximal energy of a given microRNA. For example a value of 0.25 means that the duplex has 25% of the energy of a perfect reverse complementary hybrid. In view of our intention to detect as many potential target sites as possible in the first step of our workflow we set *x *to 0.24 and *y *to 0.25, which is well below the energy cutoffs applied by other approaches [22]. On average this leads to eight candidate zones for each microRNA and 3'UTR in human (for comparison, seven sites were reported for the classic lin-4:lin-14 in *Caenorhabditis elegans *[1]).

### Duplex stacking and anchor choice

Since we do not choose duplex structures based on significant free energy values, we obtain for each microRNA-mRNA pair a vast amount of overlapping predicted secondary structures, typically in the order of 5000-20000. Therefore we need to group the resulting structures according to their location on the mRNA. Structures with a G:U match in the first 8 base pairings as well as those that show five or more G:U pairings in the entire duplex are discarded. Given that duplexes will heavily overlap on the mRNA, we need to define anchor points in order to map the duplexes to a specific position on the mRNA sequence.

Different types of anchors can be considered including, for example, the mRNA position where the first nucleotide pairing occurs, counting either from the 3' end or from the 5' end of the corresponding microRNA, or some middle point of the duplex. In view of the findings suggesting that base pairing at the 5' end of the microRNA particularly strongly contributes to target site recognition [33-37] we chose to define the anchor point as the position on the mRNA sequence where the first base-pairing with the microRNA 5' end occurs (*Additional File *5). If duplexes with a perfect microRNA 5' end pairing of at least 7 consecutive nucleotides (seed region) are present all other structures with the same anchor point and no seed region are discarded. Subsequently the energetically most favorable one for each anchor is selected as the best candidate for this specific anchor position (Figure 6A,B).

### Training set

In order to develop a classifier of high quality, it is essential to obtain a training set that is truly representative of both the positive (actual target sites) and negative (non-target sites) class. Recently a set of argonaute (Ago) - mRNA binding sites, identified by a novel technique that isolates RNA by crosslinking immunoprecipitation in high-throughput experiments (HITS-CLIP), was published for the 20 most abundant microRNAs present in the P13 mouse brain [19]. Argonautes are proteins that upon association with microRNAs form the RNA-induced silencing complex (RISC), which is responsible for the repression of target mRNA expression. To our knowledge this is the first experimental data set that reports directly microRNA target sites in a large-scale fashion and *TargetSpy *is the first algorithm that is using it for training. We retrieved the data from http://ago.rockefeller.edu/ and removed all sites that did not map to 3'UTRs or had no RefSeq accession number associated. Since only the microRNA family is specified in this publication, we identified the candidates for all microRNAs belonging to that family. Those candidates that overlapped the experimentally derived sites were retained as positive instances. In cases where several candidates of the same microRNA family overlapped, we took the energetically most favorable one. Target site candidates having no equivalent in the set of experimentally derived Ago binding sites are unlikely to be biologically relevant. We therefore identified the energetically most stable candidate for a reported Ago-mRNA interaction that does not overlap the validated Ago-binding site. Those candidates served as negative instances. In total we obtained 3872 positive and 4540 negative instances.

### Features of microRNA - mRNA duplexes

In order to build an accurate microRNA target predictor it is of paramount importance to define a set of characteristics that effectively distinguish real microRNA-mRNA interactions from any other types of hybrids. Numerous properties of such duplexes have been reported in biological literature in recent years, and some of them have been incorporated in target prediction methods developed earlier. Here, instead of relying on a limited number of empirically selected features we chose to objectively evaluate the performance of a possibly broad spectrum of pairing requirements within the framework of a machine learning approach. Below follows the list of features used in this study.

### General extent of microRNA-mRNA binding

• Number of base-pairings to the microRNA 8-mer seed.

• Number of base-pairings to the first eight nucleotides of the microRNA 3' end.

• Number of consecutive base-pairings at the microRNA 3' end with two allowed non-pairing positions, beginning at the first base pairing position.

• Length of the longest stretch of consecutive base-pairings anywhere in the hybrid.

• Length of the target site.

• Binding asymmetry. Here we measured the ratio between the amounts of paired bases in the 3' versus the 5' region of the microRNA. We considered 8 nucleotides on each side.

### Extent of G:U base pairing

• Total number of G:U wobble base pairs in the microRNA - mRNA hybrid.

### Bulge-related features of duplexes

• Number of bulges on the microRNA.

• Number of bulges on the target site.

• Total bulge length on the microRNA.

• Total bulge length on the target site.

• Number of bulges on the microRNA. We tested the bulge lengths of 1,2,3,4 and 5 bases.

• Number of bulges on the target site. In this cases we tested bulge lengths of 1,2,3,4,5,6 and equal or greater than 7 bases as bulges on the mRNA sequence tend to be larger than those on microRNAs.

• Length of the second largest bulge on the microRNA.

• Length of the second largest bulge on the target site.

• Mean length of bulges on the target site.

• Number of symmetric bulges.

### Position specific features

▪ Position of the target site in the 3'UTR. We split 3'UTRs into 100 bins and returned the index of the bin containing the anchor of the candidate zone's representative as the position of the target site.

▪ Following the reasoning of Lewis et al. [13] we calculated four features related to the base occurrence at given positions. Specifically we recorded the nucleotides in the target site at microRNA positions 1 (t1 anchor) and 9 (t9 anchor) and the existence of an S (A or U) or W (G or C) base at the same positions (t1 S/W anchor and t9 S/W anchor).

### Compositional features

Base composition of both 3' UTRs [38] and microRNAs plays an important role in mRNA-microRNA recognition. Here we employ the following compositional features:

• G + C content of the target site.

• G + C content of the 50 nucleotide long region upstream of the target site.

• G + C content of the 50 nucleotide long region downstream of the target site.

• G + C content ratio between the microRNA and the target site.

• Difference in G + C content between the target site and the upstream flanking region.

• Difference in G + C content between the first and the last eight nucleotides of the target site.

• Occurrence of CpG di-nucleotide in the target site sequence as well as in its 3' and 5' flanking regions.

The length of 3' and 5' flanking regions was taken to be 20 nucleotides, unless otherwise stated.

### Compactness

We reasoned that hybrids that are more compact, i.e. having only few unpaired nucleotides both in the microRNA and in the target site are more likely to be biologically functional that others. Therefore our goal was to unify these two features into a single measure. We define the compactness of a hybrid as the mean value of the following ratios: *number of basepairings*/*microRNA length *and *number of basepairings*/*target site length*. Compactness values are thus in the range between 0 and 1, with the latter value corresponding to perfect complementarity. If the target site is shorter than the microRNA a penalty is introduced, as this case is not taken into account by the mean of the ratios stated above:

In the equation for the compactness above, #_pair _is the number of basepairings, tsLen the target site length and miRLen the length of the microRNA.

### Accessibility of the target site to RISC

Recent literature [9,39] suggests that target site accessibility to RISC is a critical factor in microRNA target recognition. Examples of approaches that have been developed to approximate accessibility are RNAup [40] and IntaRNA [41]. We applied the definition of [9] and calculated accessibility as the difference between the free energy of the microRNA hybrid and the energy of the local secondary structure of the target site including 3 nt upstream and 15 nt downstream flanking sequences. We further tested all combinations for upstream and downstream flankings from 0 nt to 30 nt in 5 nt steps.

### Classifier

**Figure 7:**
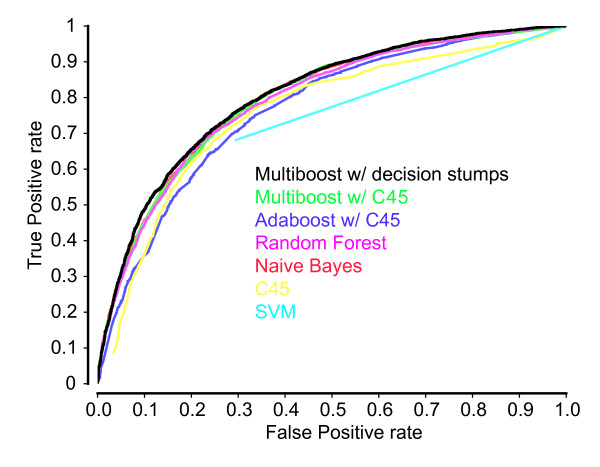
**ROC curves generated by various classifiers evaluated in 10-fold cross-validations on the training set**.

Using the positive and negative instances we developed a classifier capable of distinguishing microRNA - mRNA duplexes from other hybrids in the feature space described above. The problem is that most of the biologically motivated features implicated in microRNA target recognition, with the exception of the seed match, display only a weak correlation with functionality. A standard approach to enhance the prediction performance in case of weak features is to utilize boosting. We therefore applied the learning scheme based on boosting called MultiBoost [42] with decision stumps as base learner. In comparison with other methods we have tried (SVM [43], Naive Bayes [44], C4.5 [45], AdaBoost [46] with C4.5, MultiBoost with C4.5) it consistently produced superior results (see Figure 7). We used the WEKA [10] implementation of the learning scheme and largely relied on the standard configuration provided by WEKA, with the exception of setting the number of bagging iterations to 200. For estimating the quality of each individual feature and for subsequent ranking of features, we used the ReliefF [8] algorithm. ReliefF estimates the quality of features according to how well they distinguish between closely neighbored instances of different classes. To find the best possible set of features, we applied the feature evaluation approach called "Correlation-based Feature Selection" [11] (CFS) together with the best-first search algorithm. Only features from the subset computed by this filter approach are taken for the classifier.

### Target site prediction

For each organism considered we predicted microRNA target sites and ranked them according to their score. As explained above *TargetSpy *initially considers every potential candidate zone and assigns a score to it. Overlapping candidate zones were merged together, and the representative with the highest score becomes the representative of the entire merged zone. This permissive approach generates vast amounts of candidates with very low scores. Additional criteria are subsequently imposed in various combinations to narrow down the set of predicted targets (Table 2). The naming of the prediction datasets is based on whether or not the presence of a seed region is required, and whether a permissive (sens) or strict (spec) threshold is applied.

### Evaluation of prediction performance

For assessing the quality of our classifier we used the following performance measures: *sensitivity, specificity*, and accuracy. As in any classification process four different possibilities have to be accounted for: true positives (TP), true negatives (TN), false positives (FP) and false negatives (FN). For the evaluation on the training set, we obtained these values in form of a confusion matrix by performing a standard 10 fold cross validation followed by plotting a receiver operating characteristic (ROC) curve. From this we calculated the area under the curve (AUC) statistics, a measure that is understood as the probability that the classifier will assign a positive instance a higher score than a negative instance when picking an instance from each class randomly. Given the confusion matrix, sensitivity and specificity, are defined by the following equations:

The evaluation on pSILAC data was performed as in Selbach et al. 2008. The performance measure (accuracy) was defined as the fraction of predicted mRNA targets with reduced protein production (log_2 _fold change < -0.1), matching the definition of the positive predicted value (PPV).

### Implementation and availability

We implemented our method as a stand-alone *Java *program called *TargetSpy*. The program relies on the Java Virtual Machine version 1.5 and two freely available third party software packages - the Vienna package for RNA secondary structure prediction (version 1.6.1) and the data mining software WEKA (version 3.5.3). *TargetSpy *is available from our web site http://www.targetspy.org along with installation instructions and links to all required third party software packages.

## Authors' contributions

MS and DF conceived and directed the project. MS and MH designed the algorithm. MS implemented the algorithm. MS, MH and DL analyzed the data and evaluated the model. MS, MH and DF wrote the paper. All authors read and approved the final version.

## Supplementary Material

Additional file 1 **ROC curves, one for each class, to compare the performance of different target prediction tools**. (A) shows the evaluation of class I prediction tools, (B) of class II, and (C) of class III. Only close-ups showing the relevant area are presented. Click here for file

Additional file 2 **TargetSpy predictions for a set of 120 experimentally *tested D. melanogaster *microRNA:mRNA interactions**. Click here for file

Additional file 3 TargetSpy predictions for a set of 190 experimentally *tested D. melanogaster *microRNA:mRNA interactions. Click here for file

Additional file 4 TargetSpy predictions for a set of human microRNA:mRNA interactions with indication of whether the associated proteins show a log_2_-fold change of < -0.1. Click here for file

Additional file 5 **Defining the position of a microRNA-mRNA duplex on the mRNA sequence**. The anchor is defined as the position at which the first base pairing between the microRNA and the target site occurs, viewed from the mRNA 3' end (or microRNA 5' end). Click here for file
