# Supplementary figures and images for: TargetSpy: a supervised machine learning approach for microRNA target prediction
Source: BMC Bioinformatics. 2010 May 28;11:292. doi: 10.1186/1471-2105-11-292 (PMC2889937; doi:10.1186/1471-2105-11-292)

A

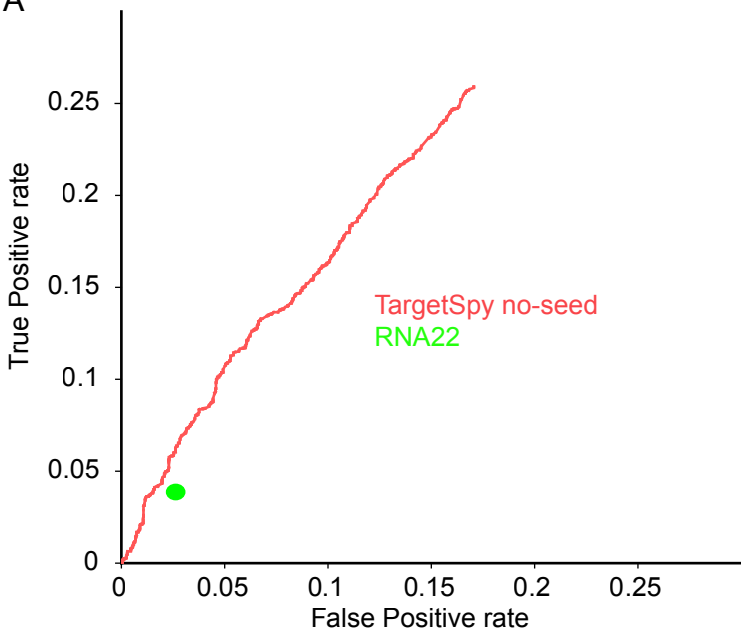

B

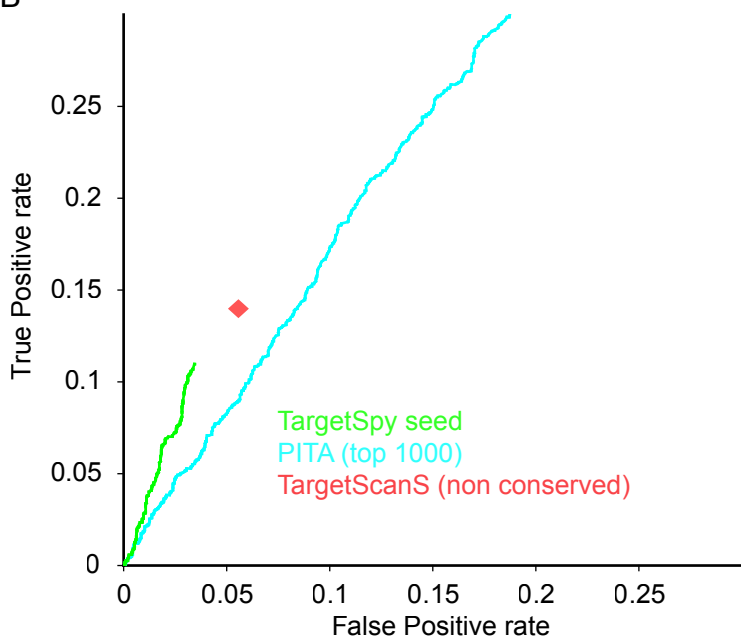

C

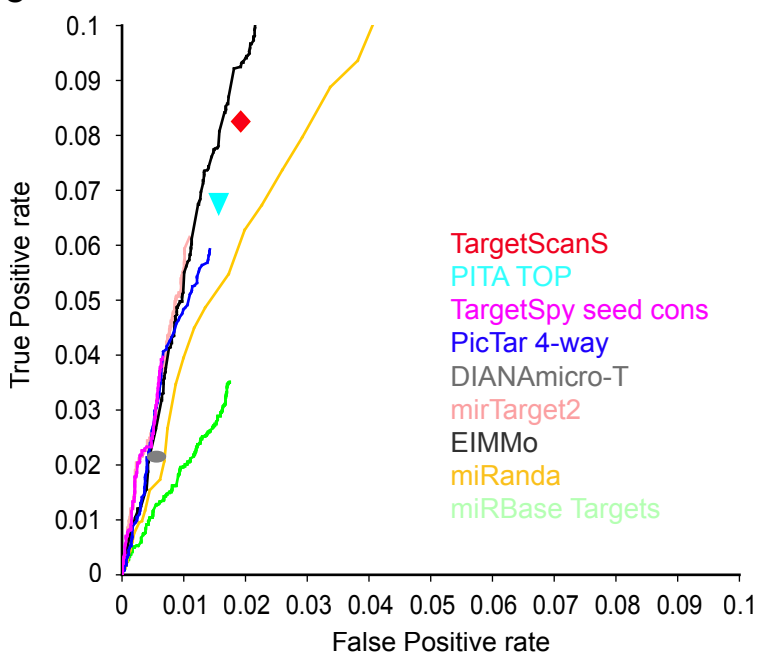

Supplement: Additional file 1 — ROC curves, one for each class, to compare the performance of different target prediction tools. (A) shows the evaluation of class I prediction tools, (B) of class II, and (C) of class III. Only close-ups showing the relevant area are presented. [file 1471-2105-11-292-S1.PDF]

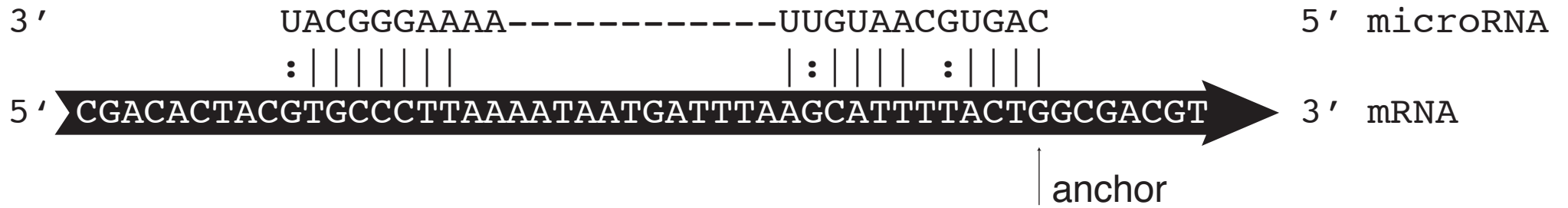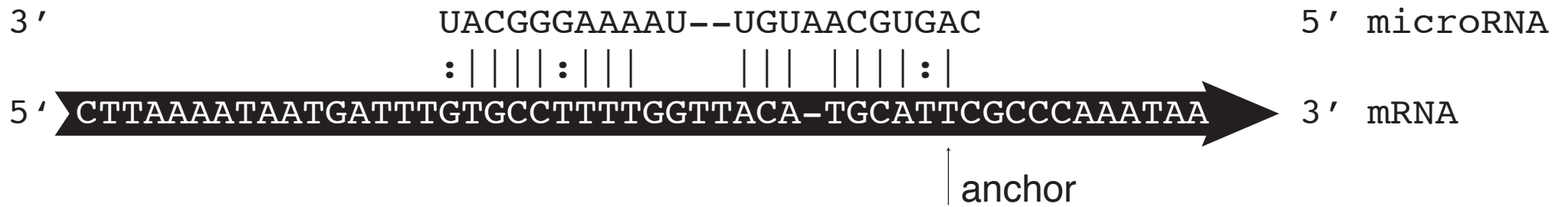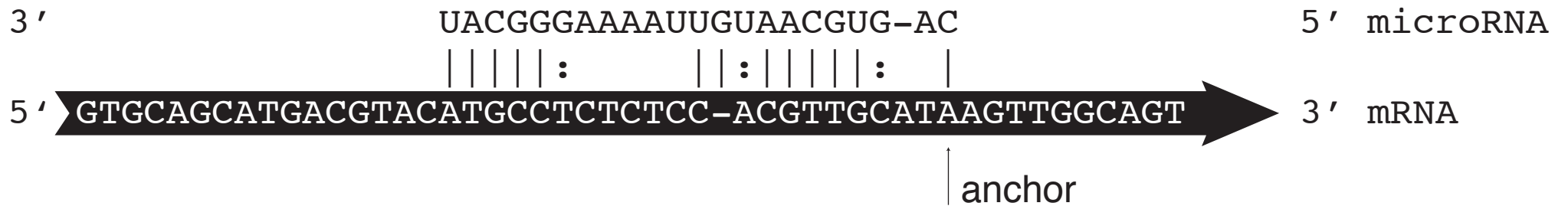

Supplement: Additional file 5 — Defining the position of a microRNA-mRNA duplex on the mRNA sequence. The anchor is defined as the position at which the first base pairing between the microRNA and the target site occurs, viewed from the mRNA 3' end (or microRNA 5' end). [file 1471-2105-11-292-S5.PDF]
